# Compensation for environmental asbestos-related diseases in South Africa: a neglected issue

**DOI:** 10.3402/gha.v6i0.19410

**Published:** 2013-01-24

**Authors:** Ntombizodwa Ndlovu, Jim teWater Naude, Jill Murray

**Affiliations:** 1 National Institute for Occupational Health, National Health Laboratory Service, Johannesburg, South Africa; 2 School of Public Health, Faculty of Health Sciences, University of the Witwatersrand, Johannesburg, South Africa; 3 Asbestos Relief Trust, Cape Town, South Africa; 4 School of Public Health, University of Cape Town, Cape Town, South Africa

**Keywords:** domestic exposure, neighbourhood exposure, pleural plaques, pleural thickening, mesothelioma, South Africa

## Abstract

**Background:**

Environmentally acquired asbestos-related diseases (ARDs) are of concern globally. In South Africa, there is widespread contamination of the environment due to historical asbestos mining operations that were poorly regulated. Although the law makes provision for the compensation of occupationally acquired ARDs, compensation for environmentally acquired ARDs is only available through the Asbestos Relief Trust (ART) and Kgalagadi Relief Trust, both of which are administered by the ART. This study assessed ARDs and compensation outcomes of environmental claims submitted to the Trusts.

**Methods:**

The personal details, medical diagnoses, and exposure information of all environmental claims considered by the Trusts from their inception in 2003 to April 2010 were used to calculate the numbers and proportions of ARDs and compensation awards.

**Results:**

There were 146 environmental claimants of whom 35 (23.9%) had fibrotic pleural disease, 1 (0.7%) had lung cancer, and 77 (52.7%) had malignant mesothelioma. 53 (36.3%) claimants were compensated: 20 with fibrotic pleural disease and 33 with mesothelioma. Of the 93 (63.7%) claimants who were not compensated, 33 had no ARDs, 18 had fibrotic pleural disease, 1 had lung cancer, and 44 had mesothelioma. In addition to having ARDs, those that were compensated had qualifying domestic (33; 62.2%) or neighbourhood (20; 37.8%) exposures to asbestos. Most of the claimants who were not compensated had ARDs but their exposures did not meet the Trusts’ exposure criteria.

**Conclusions:**

This study demonstrates the environmental impact of asbestos mining on the burden of ARDs. Mesothelioma was the most common disease diagnosed, but most cases were not compensated. This highlights that there is little redress for individuals with environmentally acquired ARDs in South Africa. To stop this ARD epidemic, there is a need for the rehabilitation of abandoned asbestos mines and the environment. These issues may not be unique to South Africa as many countries continue to mine and use asbestos.

Environmental exposures to asbestos are of concern in many parts of the world. Domestic exposures occur in people living with workers exposed to asbestos, and neighbourhood exposures occur in residents living in the vicinity of asbestos operations. Increased risks of developing mesothelioma (an asbestos related cancer) have been observed in people with no occupational exposures living near asbestos factories (1–3). Neighbourhood exposures to asbestos have been documented in Wittenoom, a former crocidolite mining region of northwest Australia (4), and Libby, Montana, where asbestos-contaminated vermiculite was mined (5, 6). True environmental asbestos exposures arise from naturally occurring asbestos contamination of the soil and have been documented in China (7), Turkey (8), Greece (9), Corsica (10), Italy (11), and New Caledonia (12), but these are not the subject of this paper.

Asbestos is a generic name for a group of fibrous hydrated silicates with commercial and industrial value because of their physical properties, which include high tensile strength, and heat and chemical resistance. South Africa is unique in that the three main commercial types of asbestos were mined there: chrysotile (white asbestos), amosite (brown asbestos), and crocidolite (blue asbestos). At the peak of production, it was the third largest producer of asbestos, with crocidolite accounting for more than 97% of global production (13). Asbestos operations generally involved families living close to the mines where men worked, while women and children cobbed, sorted, and packed fibres (14). The waste or tailings were dumped close to villages where, to this day, children play. The tailings were also used for road surfacing and building construction (13, 15). Thus, there is widespread contamination of the environment as a result of asbestos mining operations, which were scattered across rural South Africa (Fig. 1).

**Fig. 1:**
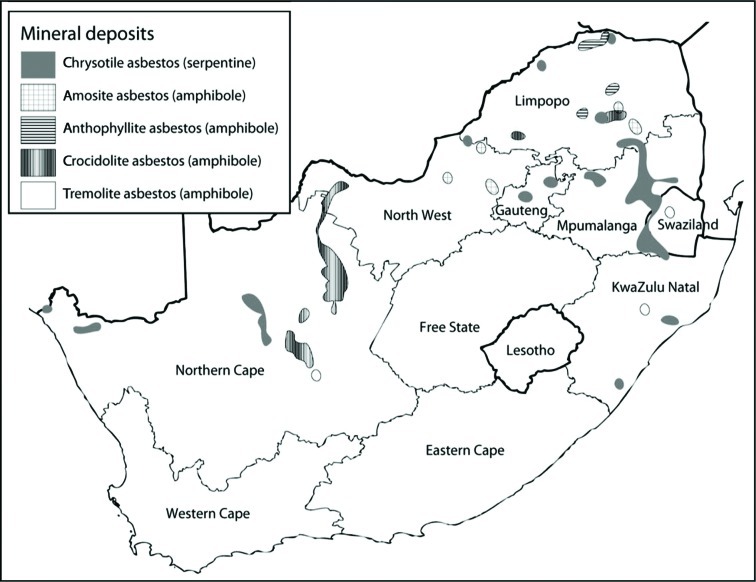
Distribution of asbestos deposits in South Africa (16).

It is generally accepted that environmental exposures are much lower, begin at younger ages and continue for longer durations than occupational exposures (17, 18). Although it is known that asbestos levels in South African mines and mills were high, data are not readily available due to poor measurement techniques and record-keeping practices at the time. Workplace amosite fibre levels ranging from less than 1 to 326.7 fibres/ml (f/ml) were measured at Penge mine (Limpopo Province) in the 1970s (19). In the Mafefe villages, to the north of Penge, much lower asbestos concentrations were measured in the 1990s: 0.020 f/ml in school buildings, 0.012 f/ml along village pathways, and 0.016 f/ml during building and gardening activities (15).

Despite the existing body of evidence on the adverse health effects of asbestos, mining continued with little enforcement of regulations until 2002 (13, 14). This has created an ‘invisible epidemic’ of asbestos-related diseases (ARDs), which include diseases of the lung parenchyma, asbestosis and lung cancer, and diseases of the pleura, such as pleural plaques, thickening, and malignant mesothelioma (20). Asbestosis and lung cancer are typically associated with high levels of asbestos exposure, which may occur in occupational settings, while pleural plaques, thickening, and malignant mesothelioma are attributed to lower level exposures. The South African landmark study that established the link between asbestos exposure and mesothelioma also identified 10 cases (30.3%) that were associated with environmental asbestos exposure (21). Other South African studies have shown environmental mesotheliomas to comprise 17–23% of ARDs in the reported study populations (22–24).

South African law compensates all occupational ARDs under the Compensation for Occupational Injuries and Diseases Act (No. 130 of 1993) (25) and, specifically for the mining industry, the Occupational Diseases in Mine and Works Act (No. 78 of 1973) (26). However, statutory redress for miners with occupational lung disease is inadequate, and dissatisfaction with very low payouts (currently ranging from R28,000 to R105,000, approximately 3,500 to 13,000 USD) has resulted in litigation. In 2003 and 2006, court settlements led to the creation of two Trusts, the Asbestos Relief Trust (ART) and Kgalagadi Relief Trust (KRT).

The ART administers the Trusts because their administrative claim procedures and benefits are essentially the same with individual claims often made against both Trusts (27). The Trusts were established primarily to compensate former workers of asbestos specific mining operations^1^ according to the provisions of their respective Trust deeds. Compensation is awarded on the basis of disease and asbestos exposure. The diseases considered are fibrotic diseases (asbestosis and/or diffuse pleural thickening and pleural plaques) and malignancies (lung cancer and mesothelioma). Fibrotic diseases are divided into those with mild to moderate lung function impairment (FEV1: 79–52% of predicted; FVC: 79–52% of predicted; FEV1/FVC: 75–55%) (ARD1) and those with severe lung function impairment (FEV1: <51%; FVC: <51%; FEV1/FVC: <55%) (ARD2).

There is no statutory compensation for environmentally acquired ARDs in South Africa; the only access to compensation for environmental claimants is through the two Trusts. The claim submission procedures for occupational and environmental claims differ. In the past, the Trusts ran awareness campaigns and actively sought potential occupational claimants throughout South Africa, Lesotho, and Swaziland. In 2011 a passive claims process was introduced, which used accredited doctors to identify and assist potential claimants with compensation submissions (29). For environmental claimants, however, the process has always been passive with the onus on each potential claimant to approach the Trusts and provide medical evidence suggestive of ARDs, including a good-quality chest X-ray obtained at the claimant's own expense (30). Full occupational and residential histories are also required.

A panel of specialist occupational medical practitioners assesses the medical information, and the Trusts pay for any additional tests. The Trusts do not ordinarily consider asbestosis and lung cancer to have been environmentally acquired because these diseases are usually attributable to high asbestos levels seen with occupational exposures (31). However, where there is compelling evidence of high and prolonged environmental exposure, the Trusts may make an award. For both occupational and environmental claimants, after an ARD diagnosis is confirmed, the exposure information is assessed for exposures that meet the Trusts’ requirements. The Trustees undertake final review and approval of the claims.

For environmental claimants, in addition to having a compensable ARD, a claimant must (i) have had domestic or neighbourhood exposure within 10 km of an asbestos work site; (ii) have been exposed during the qualifying period, 1955–2002: specific periods are applicable to each worksite; (iii) have an ARD considered by the Trusts to be caused by such exposures; and (iv) have had no occupational exposure to asbestos (30).

This study sought to determine the proportion of environmental claims submitted to the two Trusts, to describe the ARD diagnosed and, for mesothelioma cases, to document the type of asbestos to which the claimants were exposed. The study also determined the proportion of environmental claims compensated and evaluated and the efficacy of the Trusts in delivering compensation for environmentally acquired ARD.

## Methods

The design of the study is a record review of a compensation database, which employed cross-sectional passive surveillance methodology for environmental cases.

All environmental claims entered on the ART compensation database from the inception of the Trusts in 2003 to April 2010 were included. Personal details, disease diagnoses, and exposure information were obtained from the ART's database and paper records. The numbers and proportions of disease and compensation awards were assessed.

For mesothelioma cases, the likely type of asbestos exposure was assigned according to the area in which the claimant had resided (24). Crocidolite was assigned for exposures that occurred in the Northern Cape and North West Provinces, amosite for those in Limpopo, and chrysotile for those in Mpumalanga.

Ethical approval for the study was obtained from the University of the Witwatersrand Human Research Ethics Committee.

## Results

The Trusts registered a total of 15,463 compensation claims since their inception in 2003 and 2006 to April 2010. Of these, 146 (0.9%) were environmental, comprising 74 women and 72 men in the case series. Their mean ages were similar (61.8±11.9 and 56.8±11.1 years, respectively) and ranged from 21.1 to 59.3 years.

The year of first exposure to asbestos was available for 128 claimants. The exposure characteristics are summarised in Table 1. Of the 58 (45.3%) that had childhood exposures (mean 6.0±5.6 years), 20 had been exposed from birth. No significant differences in age at first exposure to asbestos, duration of exposure, or time from first exposure (latency) by disease categories were observed (data not shown).

**Table 1:** Exposure characteristics of environmental claimants

Exposure characteristic (*n*=128)	Mean±SD (years)	Range (years)
Age at first exposure	17.1±12.7	0–59
Total years of exposure	8.4±8.0	0.1–36
Time since first exposure	43.1±11.3	21–79

Thirty-three (22.6%) claimants did not have an ARD (Table 2): of these, 17 had no abnormalities visible on a chest radiograph, and the remainder had incidental findings like hyperinflation, sequelae of pulmonary tuberculosis, and scoliosis. ARDs were diagnosed in 113 (77.4%) cases. These included 35 (31.0%) with pleural thickening, most (*n*=27) of whom had mild to moderate lung function impairment (ARD1). For convenience, three cases of pleural plaques with <10% lung function impairment were added to this category. There were 78 (69.0%) claimants with malignancies of which 1 had lung cancer and 77 had mesothelioma.

**Table 2:** Asbestos-related diseases diagnosed in environmental claimants

Disease category	No.	%
Fibrotic ARD1	30*	20.5
Fibrotic ARD2	5	3.4
Lung cancer	1	0.7
Malignant mesothelioma	77	52.7
No asbestos-related diseases	33	22.6
Total	146	

* Includes 3 cases of pleural plaques with <10% lung function impairment.

Compensation outcomes are summarised in Table 3. Of all the environmental claims submitted, 53 (36.3%) were compensated as they had an ARD as well as qualifying domestic (*n*=33; 62.2%) or neighbourhood (*n*=20; 37.8%) asbestos exposures. Ninety three (63.7%) of the total claimants were not compensated. These comprised 37 who did not qualify medically: 33 had no ARD, 3 had pleural disease with <10% lung function impairment, and 1 had lung cancer. The remaining 56 claimants (12 with fibrotic ARD and 44 with mesothelioma) had exposures which did not meet the Trusts’ exposure criteria: 2 ARD2 claimants had previous occupational exposures; 4 with mesothelioma had been exposed during non-qualifying periods; 23 had lived more than 10 km from a designated operation (3 with ARD1 and 20 with mesothelioma); and the reason for non-qualifying exposure was not provided for 27 cases.

**Table 3:** Compensation outcomes for environmental claimants

	Compensated		Not compensated		Total	
			
Disease category	No.	%	No.	%	No.	%
Fibrotic ARD1	18	34.0	12	12.9	30	20.5
Fibrotic ARD2	2	3.8	3	3.2	5	3.4
Lung cancer	–	–	1	0.1	1	0.7
Malignant mesothelioma	33	62.3	44	47.3	77	52.7
No asbestos-related diseases	–	–	33	35.5	33	22.6
Total	53		93		146	

Mesothelioma was diagnosed in 34 (44.2%) women and 43 (55.8%) men, and their mean ages were similar (60.9±11.1 and 58.2±9.8 years, respectively). Most mesothelioma cases with known exposures had been exposed to crocidolite asbestos (*n*=70; 90.9%) (Table 4).

**Table 4:** Geographic origin and asbestos fibre types of environmental mesothelioma claimants

Province	Likely fibre type	No.	%
Northern Cape	Crocidolite	64	81.8
North West	Crocidolite	6	7.8
Limpopo	Amosite	1	1.3
Unknown	Unknown	6	7.8
Total		77	

## Discussion

This study highlights the contribution of environmental asbestos exposure to the burden of ARDs. The environmental claims reviewed comprised 0.9% of the total claims made to the Trusts. However, this may be an underestimate as the claim process may preclude many potential environmental claimants who are required to provide evidence of disease which may be costly. This case-series under-ascertains the extent of ARD as the analysis was based on submissions made to the Trusts, which have limited geographical coverage, and migration may result in many potential claimants moving out of the Trusts’ target areas. The under-diagnosis of ARDs in public health care facilities, where clinicians may be unaware of or ill equipped to diagnose these diseases, also contributes to under-ascertainment (32). In response, the Trusts partnered with the National Institute for Occupational Health and conducted ARD diagnosis workshops for health-care practitioners in affected areas. It can be assumed, therefore, that more extensive awareness campaigns, improved diagnosis, and active case finding will identify many more environmentally acquired ARDs.

Fibrotic pleural ARD accounted for 23.9% and mesothelioma for approximately half (52.7%) of the claims. While lung function impairment associated with fibrotic pleural ARD may cause significant disability, the impact of mesothelioma is particularly devastating as there is no satisfactory clinical treatment and the prognosis is poor, with survival typically less than 18 months after diagnosis (17). Almost all of the reported mesothelioma cases had been exposed to crocidolite asbestos. In South Africa, crocidolite has been shown to be the most frequent cause of mesothelioma even after brief or slight exposures (22, 33). It is notable that many exposures had occurred in childhood.

Mesothelioma is an indicator of the ARD burden of a population. The burden is currently borne predominantly by the developed world and varies among countries. A shift in the burden is expected as some developing countries increase their use of asbestos (34). Around the year 2000, high crude annual incidence estimates of 30 cases per million were reported in Australia, Belgium, and Great Britain (35). South Africa reported 2,322 mesothelioma deaths from 1994 to 2008, which accounted for 2.5% of the reported mesothelioma deaths and ranking it ninth among the 83 countries studied (34). In 1984, the age-adjusted mesothelioma incidence rates in South Africa ranged from 3.2 per million/year in black women to 40.5 in white men (23). In 2007, the reported overall mortality rates were much lower than expected at 13 and 3 per million/year for men and women, respectively (36). The decline was attributed to migration, decreased life expectancy due to other causes, and long latency. However, it is projected that the improved life expectancy in Africa will be accompanied by an increase in the burden of diseases with long latency, such as mesothelioma (37).

Compensation payouts for ARDs provide significant financial respite to families and communities in the former asbestos mining areas of South Africa, which are characterised by desperate economic circumstances and high levels of unemployment (38). Approximately two-thirds (*n*=93, 63.7%) of the environmental claims reviewed here were not compensated. Many had been exposed to asbestos more than 10 km from designated mining operations, and some had exposures that occurred outside of the qualifying periods. In the absence of complete rehabilitation of the dumps, many more cases of environmentally acquired ARDs that do not meet the Trusts’ exposure criteria are to be expected.

The findings of this study support the argument that the Trusts should review their exposure criteria. The mining operations that led to their creation contributed significantly to contamination of the environment in many regions; for example, in the Kuruman area the Trusts represent companies that generated over 85% of the tonnage of asbestos mined in this area (Jim teWater Naude, personal communication, 10 October 2012). At the time that the environmental compensation criteria were set out by the Trusts, a radius of 10 km from the source of contamination was adopted in the light of preliminary work done by Jones and subsequently published in his thesis on environmental asbestos contamination in South Africa (39). In his discussion, however, Jones makes the point that no clear linear correlation can be drawn between distance from a source point and levels of disease. In addition, there are many asbestos contaminated areas in South Africa where the sources of contamination are not included in the remit of the Trusts because the founders of the Trusts were never active in these regions. The State has a duty to accept responsibility for these areas, and it has been argued that the State should use some of the considerable revenue that it received from asbestos mining to compensate environmentally acquired ARDs and to ensure that contaminated areas are rehabilitated.

## Conclusion

The cessation of asbestos mining and use in South Africa has drastically reduced occupational exposures, but widespread contamination of the environment, from decades of production, suggests an undefined and perhaps extensive ARD epidemic. While compensation systems exist for occupationally acquired ARDs, this study demonstrates that there is very little redress for people with environmentally acquired disease.

This study highlights that contamination of the environment and domestic exposures to asbestos contribute to the burden of ARDs. It is therefore important that workers exposed to asbestos and communities in the vicinity of asbestos operations are made aware of the risks associated with domestic exposures and strategies to prevent contamination of the environment. A recent global policy framework recommends prevention of occupational and environmental cancers through education, standardisation and enforcement of regulations, and disease surveillance (40).

Calls have been made for the South African government to address asbestos-related pollution of the environment (15, 41). Many dumps have been rehabilitated, but erosion of the rehabilitated dumps and exposure to the tailings that were used for road and building construction by nearby communities remain issues that need to be addressed. Improved monitoring and evaluation is required to ensure that exposures remain low and that interventions that are implemented are sustained (42).

Historically, the focus has been on occupationally acquired ARDs in former asbestos mine, mill, and factory workers. A lesson from this study is that exposure to asbestos outside the workplace contributes to the burden of ARDs and should not be ignored. It is likely that most future ARDs in South Africa will result from neighbourhood exposures as men, women, and children continue to be inadvertently exposed to asbestos. In light of this study, urgent attention needs to be directed towards dealing with compensation for environmental mesotheliomas.

## Footnotes

1 The ART administers compensation on behalf of claimants against Griqualand Exploration and Finance Company (Gefco), General Mining Corporation (Gencor), Msauli, African Chrysotile Asbestos Limited (ACA), Hanova Mining Holdings, and Cape PLC. Similarly, the KRT was established for individuals who worked at the Kuruman Cape Blue (KCBA) and Danielskuil Cape Blue (DCBA) asbestos mines (28).
